# Poly[[μ_2_-(1*Z*,*N*′*E*)-2-(1,3-benzothia­zol-2-ylsulfan­yl)-*N*′-(2-oxidobenzyl­idene-κ^2^ *O*:*O*)acetohydrazidato-κ^2^ *O*,*N*′](pyridine-κ*N*)copper(II)]

**DOI:** 10.1107/S160053681004986X

**Published:** 2010-12-04

**Authors:** Vladimir V. Bon, Svitlana I. Orysyk, Vasily I. Pekhnyo

**Affiliations:** a Institute of General and Inorganic Chemistry, NAS Ukraine, Kyiv, Prosp. Palladina 32/34, 03680, Ukraine

## Abstract

In the title compound, [Cu(C_16_H_11_N_3_O_2_S_2_)(C_5_H_5_N)]_*n*_, the Cu^II^ atom displays a square-pyramidal CuN_2_O_3_ coordination geometry with strong elongation in the vertex direction. The hydrazone mol­ecule is coordinated to the Cu^II^ atom in a tridentate manner in the enolic form, creating five- and six-membered chelate metallarings. The pyridine mol­ecule completes the square-planar base of the copper coordination environment. The crystal structure displays zigzag polymeric Cu—O—Cu chains along [001]. Several weak π–π inter­actions between benzothia­zole rings were found in the same direction [centroid–centroid distances = 3.7484 (16), 3.7483 (16), 3.6731 (17) and 3.7649 (17) Å].

## Related literature

For general background to the biological activity of hydrazones and their metal complexes, see: Belkheiri *et al.* (2010 ▶); Pavan *et al.* (2010 ▶). For related structures, see: Luo *et al.* (2009 ▶).
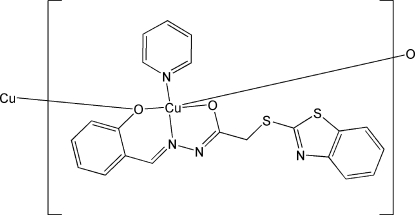


## Experimental

### 

#### Crystal data

[Cu(C_16_H_11_N_3_O_2_S_2_)(C_5_H_5_N)]
*M* *_r_* = 484.04
Orthorhombic, 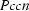

*a* = 21.6256 (5) Å
*b* = 25.3751 (7) Å
*c* = 7.1230 (2) Å
*V* = 3908.76 (18) Å^3^
*Z* = 8
Mo *K*α radiation
μ = 1.36 mm^−1^
*T* = 173 K
0.50 × 0.08 × 0.06 mm

#### Data collection

Bruker APEXII CCD diffractometer
Absorption correction: multi-scan (*SADABS*; Bruker, 2005 ▶) *T*_min_ = 0.550, *T*_max_ = 0.923
18118 measured reflections
4003 independent reflections
2903 reflections with *I* > 2σ(*I*)
*R*_int_ = 0.055

#### Refinement

*R*[*F* ^2^ > 2σ(*F* ^2^)] = 0.037
*wR*(*F* ^2^) = 0.089
*S* = 1.02
4003 reflections
271 parameters
H-atom parameters constrained
Δρ_max_ = 0.38 e Å^−3^
Δρ_min_ = −0.33 e Å^−3^

### 

Data collection: *APEX2* (Bruker, 2005 ▶); cell refinement: *SAINT* (Bruker, 2005 ▶); data reduction: *SAINT*; program(s) used to solve structure: *SHELXS97* (Sheldrick, 2008 ▶); program(s) used to refine structure: *SHELXL97* (Sheldrick, 2008 ▶); molecular graphics: *DIAMOND* (Brandenburg & Putz, 2010 ▶); software used to prepare material for publication: *publCIF* (Westrip, 2010 ▶).

## Supplementary Material

Crystal structure: contains datablocks I, global. DOI: 10.1107/S160053681004986X/rk2250sup1.cif

Structure factors: contains datablocks I. DOI: 10.1107/S160053681004986X/rk2250Isup2.hkl

Additional supplementary materials:  crystallographic information; 3D view; checkCIF report

## Figures and Tables

**Fig. 1.**
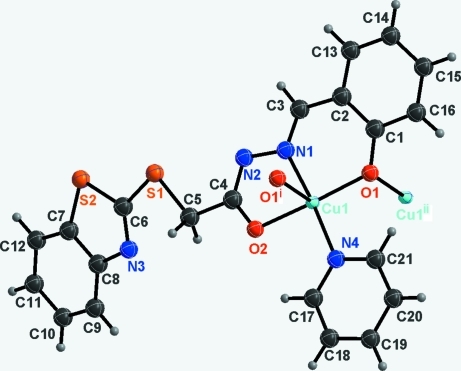
Asymmetric unit of title compound with the atom numbering scheme. Displacement ellipsoids are shown at 50% probability level. H atoms are presented as a small spheres of arbitrary radius. Atoms O1i and Cu1ii generated using symmetry operators: (i) 3/2-x, y, -1/2+z; (ii) 3/2-x, y, 1/2+z.

**Fig. 2.**
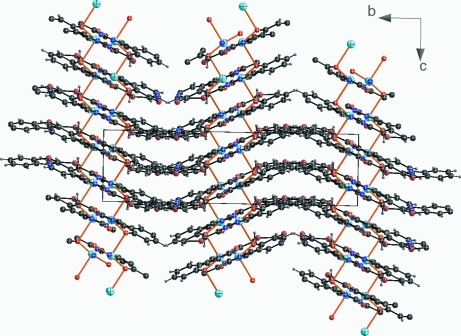
Crystal structure of title compound. View along a axis.

## supplementary crystallographic information

### Comment

Structure investigation of hydrazones and their metal complexes attract an interest due to their antioxidant, antimycobacterium, antituberculosis activity and cytotoxicity (Belkheiri *et al.*, 2010; Pavan *et al.*, 2010). In the current paper we report the structure investigation of copper(II) one–dimensional coordination polymer obtained at room temperature.

The asymmetric unit of title compound contains one monomeric chain of the polymer. Copper atom displays square–pyramidal CuN_2_O_3_ coordination geometry, which is strongly elongated in vertex direction (Fig. 1). Hydrazone molecule is coordinated tridentantly in double deprotonated enolic form creating five– and six–membered chelate metalla rings. The square–planar base of copper coordination polyhedron is complemented by coordinated pyridine molecule. The values of Cu—O and Cu—N bond lengths corresponds to related structures (Luo *et al.*, 2009). The geometry of coordination polyhedron is slightly distorted with adjacent angles in the range 80.71 (9)–99.22 (8)°. The Cu–Cu distance between two copper atoms in the polymeric chain is 3.5640 (5)Å. The valence angles O1–Cu1–O1^i^ and Cu1–O1–Cu1^ii^ have a same value of 99.22 (8)°. Symmetry codes: (i) 3/2-*x*, *y*, -1/2+*z*; (ii) 3/2-*x*, *y*, 1/2+*z*. The five–membered metalla ring Cu1/O2/C4/N2/N1 has a planar geometry with mean deviation from plane 0.0327Å. Contrariwise, the six–membered ring has an envelope conformation with dihedral angle 22.60 (15)° between Cu1/O1/N1 and O1/C1/C2/C3/N1 planes. The pyridine ring is nearly coplanar to square–planar base of copper coordination environment. The dihedral angle between planes Cu1/O1/O2/N1/N2 and N2/C17—C21 creates 11.02 (14)°. Crystal structure of title compound displays 1D zigzag chains (Cu–O–Cu) along [001] direction (Fig. 2). The adjacent polymeric chains in the crystal structure are connected by weak π–π stacking interactions between benzthiazol and phenyl rings: *Cg*1···*Cg*1^iii^ = 3.7484 (16)Å, α = 3°; *Cg*1···*Cg*1^iv^ =  3.7483 (16)Å, α = 3°; *Cg*1···*Cg*2^iii^ = 3.6731 (17)Å, α = 2.18 (14)°; *Cg*1···*Cg*2^iv^  = 3.7649 (17)Å, α = 2.18 (14)°; (*Cg*1 - centroid of the ring S2/N3/C6/C7/C8, *Cg*2 – centroid of the ring C7–C12, α - dihedral angle between rings. Symmetry codes: (iii) *x*, 1/2-*y*, -1/2+*z*; (iv) *x*, 1/2-*y*, 1/2+*z*.

### Experimental

Mixture of 20 ml (10 ^-2^ mol/*L*) aqueous solution of copper(II) acetate with 2 ml of pyridine was stirred with 20 ml (10 ^-2^ mol/*L*) ethanolic solution of (*E*)-2-(benzo[*d*]thiazol-2-ylthio)-*N*'-(2-\ hydroxybenzylidene)acetohydrazide for 1 h. The resulted solution was leaved in dark place for evaporation. After 1 week of stating violet needle–like shape crystals were grown.

### Refinement

The positions of all H atoms were calculated regarding with hybridization of the parent atom and refined using riding model with d(C—H) = 0.99Å for CH_2_, d(C—H) = 0.95Å for CH with *U*_iso_(H) = 1.2*U*_eq_(C).

### Crystal data

**Table:**

[Cu(C_16_H_11_N_3_O_2_S_2_)(C_5_H_5_N)]	*D*_x_ = 1.645 Mg m^−^^3^
*M**_r_* = 484.04	Melting point: 553 K
Orthorhombic, *P**c**c**n*	Mo *K*α radiation, λ = 0.71073 Å
Hall symbol: -P 2ab 2ac	Cell parameters from 2558 reflections
*a* = 21.6256 (5) Å	θ = 2.5–25.0°
*b* = 25.3751 (7) Å	µ = 1.36 mm^−^^1^
*c* = 7.1230 (2) Å	*T* = 173 K
*V* = 3908.76 (18) Å^3^	Needle, violet
*Z* = 8	0.50 × 0.08 × 0.06 mm
*F*(000) = 1976	

### Data collection

**Table:**

Bruker APEXII CCD diffractometer	4003 independent reflections
Radiation source: fine-focus sealed tube	2903 reflections with *I* > 2σ(*I*)
graphite	*R*_int_ = 0.055
φ and ω scans	θ_max_ = 26.4°, θ_min_ = 1.9°
Absorption correction: multi-scan (*SADABS*; Bruker, 2005)	*h* = −27→24
*T*_min_ = 0.550, *T*_max_ = 0.923	*k* = −27→31
18118 measured reflections	*l* = −8→8

### Refinement

**Table:**

Refinement on *F*^2^	Primary atom site location: structure-invariant direct methods
Least-squares matrix: full	Secondary atom site location: difference Fourier map
*R*[*F*^2^ > 2σ(*F*^2^)] = 0.037	Hydrogen site location: inferred from neighbouring sites
*wR*(*F*^2^) = 0.089	H-atom parameters constrained
*S* = 1.02	*w* = 1/[σ^2^(*F*_o_^2^) + (0.0326*P*)^2^ + 3.0387*P*] where *P* = (*F*_o_^2^ + 2*F*_c_^2^)/3
4003 reflections	(Δ/σ)_max_ = 0.001
271 parameters	Δρ_max_ = 0.38 e Å^−^^3^
0 restraints	Δρ_min_ = −0.33 e Å^−^^3^

### Special details

**Table:**

Geometry. All s.u.'s (except the s.u. in the dihedral angle between two l.s. planes) are estimated using the full covariance matrix. The cell s.u.'s are taken into account individually in the estimation of s.u.'s in distances, angles and torsion angles; correlations between s.u.'s in cell parameters are only used when they are defined by crystal symmetry. An approximate (isotropic) treatment of cell s.u.'s is used for estimating s.u.'s involving l.s. planes.
Refinement. Refinement of *F*^2^ against ALL reflections. The weighted *R*–factor *wR* and goodness of fit *S* are based on *F*^2^, conventional *R*–factors *R* are based on *F*, with *F* set to zero for negative *F*^2^. The threshold expression of *F*^2^ > σ(*F*^2^) is used only for calculating *R*–factors(gt) *etc*. and is not relevant to the choice of reflections for refinement. *R*–factors based on *F*^2^ are statistically about twice as large as those based on *F*, and *R*–factors based on ALL data will be even larger.

### Fractional atomic coordinates and isotropic or equivalent isotropic displacement parameters (Å^2^)

**Table:**

	*x*	*y*	*z*	*U*_iso_*/*U*_eq_	
Cu1	0.753102 (15)	−0.037321 (13)	0.25835 (5)	0.02055 (11)	
S1	0.60090 (3)	0.11100 (3)	−0.02890 (11)	0.02363 (19)	
S2	0.53776 (3)	0.21393 (3)	−0.09219 (12)	0.02583 (19)	
N1	0.66488 (10)	−0.03048 (9)	0.2427 (3)	0.0187 (5)	
N2	0.64317 (10)	0.01688 (9)	0.1648 (4)	0.0200 (5)	
N3	0.65432 (11)	0.20772 (9)	−0.0010 (4)	0.0237 (6)	
N4	0.84545 (10)	−0.04057 (9)	0.2569 (3)	0.0205 (5)	
O1	0.74669 (8)	−0.09381 (8)	0.4338 (3)	0.0256 (5)	
O2	0.74812 (8)	0.02913 (7)	0.1199 (3)	0.0251 (5)	
C1	0.69993 (13)	−0.12735 (11)	0.4391 (4)	0.0215 (7)	
C2	0.64019 (12)	−0.11621 (11)	0.3652 (4)	0.0204 (6)	
C3	0.62496 (13)	−0.06622 (11)	0.2816 (4)	0.0207 (7)	
H3	0.5828	−0.0593	0.2535	0.025*	
C4	0.69083 (12)	0.04394 (11)	0.1080 (4)	0.0194 (6)	
C5	0.68084 (12)	0.09696 (11)	0.0213 (5)	0.0246 (7)	
H5A	0.7048	0.0990	−0.0969	0.030*	
H5B	0.6970	0.1243	0.1074	0.030*	
C6	0.60595 (13)	0.17951 (11)	−0.0371 (4)	0.0213 (7)	
C7	0.57858 (13)	0.27236 (11)	−0.0661 (4)	0.0222 (7)	
C8	0.63975 (13)	0.26134 (11)	−0.0164 (4)	0.0225 (7)	
C9	0.68083 (14)	0.30265 (12)	0.0145 (5)	0.0282 (7)	
H9	0.7226	0.2958	0.0483	0.034*	
C10	0.65983 (14)	0.35356 (12)	−0.0049 (5)	0.0295 (7)	
H10	0.6874	0.3821	0.0165	0.035*	
C11	0.59887 (14)	0.36401 (12)	−0.0552 (4)	0.0270 (7)	
H11	0.5857	0.3995	−0.0680	0.032*	
C12	0.55732 (13)	0.32394 (12)	−0.0868 (4)	0.0247 (7)	
H12	0.5157	0.3311	−0.1214	0.030*	
C13	0.59384 (13)	−0.15471 (12)	0.3780 (5)	0.0291 (8)	
H13	0.5541	−0.1475	0.3272	0.035*	
C14	0.60447 (15)	−0.20236 (13)	0.4619 (5)	0.0371 (9)	
H14	0.5727	−0.2282	0.4666	0.045*	
C15	0.66156 (15)	−0.21258 (13)	0.5395 (5)	0.0382 (9)	
H15	0.6688	−0.2453	0.6002	0.046*	
C16	0.70815 (14)	−0.17588 (12)	0.5300 (5)	0.0313 (8)	
H16	0.7469	−0.1836	0.5865	0.038*	
C17	0.87917 (12)	−0.00032 (11)	0.1905 (4)	0.0221 (7)	
H17	0.8584	0.0293	0.1384	0.027*	
C18	0.94300 (13)	−0.00042 (12)	0.1953 (5)	0.0273 (7)	
H18	0.9654	0.0287	0.1463	0.033*	
C19	0.97394 (14)	−0.04243 (12)	0.2708 (4)	0.0294 (8)	
H19	1.0178	−0.0426	0.2788	0.035*	
C20	0.93942 (13)	−0.08465 (12)	0.3352 (5)	0.0271 (7)	
H20	0.9594	−0.1149	0.3850	0.033*	
C21	0.87554 (13)	−0.08248 (12)	0.3266 (4)	0.0239 (7)	
H21	0.8522	−0.1116	0.3715	0.029*	

### Atomic displacement parameters (Å^2^)

**Table:**

	*U*^11^	*U*^22^	*U*^33^	*U*^12^	*U*^13^	*U*^23^
Cu1	0.01372 (17)	0.02016 (19)	0.0278 (2)	−0.00024 (15)	−0.00072 (15)	0.00473 (17)
S1	0.0198 (4)	0.0180 (4)	0.0331 (5)	0.0017 (3)	−0.0009 (3)	0.0003 (3)
S2	0.0218 (4)	0.0219 (4)	0.0337 (5)	0.0014 (3)	−0.0039 (3)	0.0008 (4)
N1	0.0177 (12)	0.0178 (13)	0.0207 (13)	0.0016 (9)	0.0006 (10)	0.0018 (11)
N2	0.0203 (12)	0.0161 (12)	0.0235 (14)	0.0037 (10)	0.0000 (10)	0.0000 (11)
N3	0.0259 (13)	0.0174 (13)	0.0277 (15)	0.0019 (10)	−0.0008 (11)	−0.0019 (12)
N4	0.0175 (12)	0.0225 (13)	0.0214 (14)	0.0007 (10)	−0.0025 (10)	−0.0027 (11)
O1	0.0198 (10)	0.0220 (11)	0.0349 (13)	−0.0019 (8)	−0.0029 (9)	0.0093 (10)
O2	0.0160 (10)	0.0221 (11)	0.0372 (13)	0.0000 (8)	0.0009 (9)	0.0070 (10)
C1	0.0224 (15)	0.0187 (16)	0.0234 (17)	0.0005 (12)	0.0056 (12)	−0.0024 (13)
C2	0.0185 (14)	0.0203 (15)	0.0224 (17)	−0.0019 (12)	0.0039 (12)	−0.0012 (13)
C3	0.0157 (14)	0.0227 (16)	0.0236 (18)	−0.0006 (11)	0.0002 (12)	−0.0032 (13)
C4	0.0208 (14)	0.0191 (15)	0.0183 (15)	0.0019 (12)	0.0000 (12)	−0.0038 (13)
C5	0.0169 (14)	0.0205 (16)	0.036 (2)	0.0009 (11)	0.0047 (13)	0.0048 (14)
C6	0.0222 (15)	0.0196 (16)	0.0221 (17)	0.0031 (12)	0.0010 (12)	−0.0007 (13)
C7	0.0233 (15)	0.0247 (16)	0.0185 (16)	−0.0009 (12)	−0.0004 (12)	0.0002 (14)
C8	0.0224 (15)	0.0205 (17)	0.0247 (17)	0.0035 (11)	0.0011 (13)	−0.0010 (13)
C9	0.0235 (15)	0.0271 (17)	0.034 (2)	−0.0004 (13)	−0.0044 (14)	−0.0029 (15)
C10	0.0317 (17)	0.0223 (17)	0.035 (2)	−0.0039 (13)	0.0018 (14)	−0.0022 (15)
C11	0.0355 (17)	0.0188 (16)	0.0266 (18)	0.0073 (13)	0.0027 (14)	0.0030 (14)
C12	0.0239 (15)	0.0278 (17)	0.0223 (17)	0.0061 (12)	−0.0007 (13)	0.0034 (15)
C13	0.0216 (15)	0.0272 (18)	0.039 (2)	−0.0052 (13)	0.0017 (14)	−0.0050 (16)
C14	0.0324 (18)	0.0233 (18)	0.056 (3)	−0.0090 (14)	0.0096 (16)	0.0027 (17)
C15	0.0387 (19)	0.0196 (17)	0.056 (3)	0.0029 (14)	0.0136 (17)	0.0086 (17)
C16	0.0268 (16)	0.0256 (18)	0.041 (2)	0.0035 (13)	0.0063 (15)	0.0091 (16)
C17	0.0205 (14)	0.0185 (15)	0.0274 (17)	0.0001 (12)	0.0019 (12)	−0.0024 (13)
C18	0.0205 (15)	0.0271 (18)	0.0341 (19)	−0.0011 (13)	0.0030 (13)	−0.0019 (15)
C19	0.0186 (15)	0.040 (2)	0.0300 (19)	0.0041 (13)	0.0023 (13)	−0.0022 (16)
C20	0.0246 (16)	0.0266 (17)	0.0302 (18)	0.0081 (13)	−0.0035 (14)	−0.0011 (15)
C21	0.0245 (15)	0.0236 (17)	0.0236 (17)	0.0014 (13)	0.0012 (13)	−0.0005 (14)

### Geometric parameters (Å, °)

**Table:**

Cu1—O1	1.906 (2)	C7—C12	1.395 (4)
Cu1—N1	1.919 (2)	C7—C8	1.398 (4)
Cu1—O2	1.9566 (19)	C8—C9	1.391 (4)
Cu1—N4	1.999 (2)	C9—C10	1.376 (4)
Cu1—O1^i^	2.720 (2)	C9—H9	0.9500
S1—C6	1.743 (3)	C10—C11	1.392 (4)
S1—C5	1.801 (3)	C10—H10	0.9500
S2—C7	1.736 (3)	C11—C12	1.375 (4)
S2—C6	1.758 (3)	C11—H11	0.9500
N1—C3	1.282 (3)	C12—H12	0.9500
N1—N2	1.405 (3)	C13—C14	1.368 (4)
N2—C4	1.303 (3)	C13—H13	0.9500
N3—C6	1.293 (4)	C14—C15	1.377 (5)
N3—C8	1.401 (4)	C14—H14	0.9500
N4—C17	1.341 (4)	C15—C16	1.374 (4)
N4—C21	1.342 (4)	C15—H15	0.9500
O1—C1	1.322 (3)	C16—H16	0.9500
O2—C4	1.298 (3)	C17—C18	1.381 (4)
C1—C16	1.403 (4)	C17—H17	0.9500
C1—C2	1.423 (4)	C18—C19	1.369 (4)
C2—C13	1.403 (4)	C18—H18	0.9500
C2—C3	1.440 (4)	C19—C20	1.384 (4)
C3—H3	0.9500	C19—H19	0.9500
C4—C5	1.496 (4)	C20—C21	1.384 (4)
C5—H5A	0.9900	C20—H20	0.9500
C5—H5B	0.9900	C21—H21	0.9500
			
O1—Cu1—N1	91.94 (9)	C12—C7—S2	128.5 (2)
O1—Cu1—O2	167.00 (9)	C8—C7—S2	109.7 (2)
N1—Cu1—O2	80.68 (9)	C9—C8—C7	119.6 (3)
O1—Cu1—N4	92.59 (9)	C9—C8—N3	125.1 (3)
N1—Cu1—N4	175.39 (10)	C7—C8—N3	115.3 (3)
O2—Cu1—N4	95.04 (9)	C10—C9—C8	118.7 (3)
O1—Cu1—O1^i^	99.26 (8)	C10—C9—H9	120.6
N1—Cu1—O1^i^	89.99 (8)	C8—C9—H9	120.6
O2—Cu1—O1^i^	91.47 (8)	C9—C10—C11	121.1 (3)
N4—Cu1—O1^i^	88.40 (8)	C9—C10—H10	119.4
C6—S1—C5	98.27 (13)	C11—C10—H10	119.4
C7—S2—C6	88.50 (14)	C12—C11—C10	121.3 (3)
C3—N1—N2	117.7 (2)	C12—C11—H11	119.3
C3—N1—Cu1	126.36 (19)	C10—C11—H11	119.3
N2—N1—Cu1	115.64 (16)	C11—C12—C7	117.4 (3)
C4—N2—N1	108.0 (2)	C11—C12—H12	121.3
C6—N3—C8	109.9 (2)	C7—C12—H12	121.3
C17—N4—C21	118.1 (2)	C14—C13—C2	121.6 (3)
C17—N4—Cu1	120.92 (19)	C14—C13—H13	119.2
C21—N4—Cu1	121.00 (19)	C2—C13—H13	119.2
C1—O1—Cu1	123.95 (19)	C13—C14—C15	119.5 (3)
C4—O2—Cu1	109.57 (17)	C13—C14—H14	120.3
O1—C1—C16	118.8 (3)	C15—C14—H14	120.3
O1—C1—C2	123.8 (3)	C16—C15—C14	120.7 (3)
C16—C1—C2	117.4 (3)	C16—C15—H15	119.7
C13—C2—C1	119.1 (3)	C14—C15—H15	119.7
C13—C2—C3	118.5 (3)	C15—C16—C1	121.7 (3)
C1—C2—C3	122.4 (2)	C15—C16—H16	119.2
N1—C3—C2	123.9 (3)	C1—C16—H16	119.2
N1—C3—H3	118.0	N4—C17—C18	122.2 (3)
C2—C3—H3	118.0	N4—C17—H17	118.9
O2—C4—N2	125.6 (3)	C18—C17—H17	118.9
O2—C4—C5	115.2 (2)	C19—C18—C17	120.0 (3)
N2—C4—C5	119.2 (2)	C19—C18—H18	120.0
C4—C5—S1	113.48 (19)	C17—C18—H18	120.0
C4—C5—H5A	108.9	C18—C19—C20	118.0 (3)
S1—C5—H5A	108.9	C18—C19—H19	121.0
C4—C5—H5B	108.9	C20—C19—H19	121.0
S1—C5—H5B	108.9	C21—C20—C19	119.5 (3)
H5A—C5—H5B	107.7	C21—C20—H20	120.2
N3—C6—S1	126.6 (2)	C19—C20—H20	120.2
N3—C6—S2	116.6 (2)	N4—C21—C20	122.1 (3)
S1—C6—S2	116.77 (16)	N4—C21—H21	118.9
C12—C7—C8	121.8 (3)	C20—C21—H21	118.9
			
O1—Cu1—N1—C3	−22.8 (3)	C6—S1—C5—C4	158.5 (2)
O2—Cu1—N1—C3	168.0 (3)	C8—N3—C6—S1	−178.0 (2)
O1^i^—Cu1—N1—C3	76.5 (2)	C8—N3—C6—S2	−0.3 (3)
O1—Cu1—N1—N2	163.35 (19)	C5—S1—C6—N3	−4.6 (3)
O2—Cu1—N1—N2	−5.89 (19)	C5—S1—C6—S2	177.81 (18)
O1^i^—Cu1—N1—N2	−97.38 (19)	C7—S2—C6—N3	0.6 (3)
C3—N1—N2—C4	−169.8 (3)	C7—S2—C6—S1	178.43 (19)
Cu1—N1—N2—C4	4.6 (3)	C6—S2—C7—C12	180.0 (3)
O1—Cu1—N4—C17	−163.1 (2)	C6—S2—C7—C8	−0.6 (2)
O2—Cu1—N4—C17	6.4 (2)	C12—C7—C8—C9	0.3 (5)
O1^i^—Cu1—N4—C17	97.7 (2)	S2—C7—C8—C9	−179.1 (2)
O1—Cu1—N4—C21	15.2 (2)	C12—C7—C8—N3	−180.0 (3)
O2—Cu1—N4—C21	−175.4 (2)	S2—C7—C8—N3	0.6 (3)
O1^i^—Cu1—N4—C21	−84.0 (2)	C6—N3—C8—C9	179.5 (3)
N1—Cu1—O1—C1	29.8 (2)	C6—N3—C8—C7	−0.2 (4)
O2—Cu1—O1—C1	84.8 (4)	C7—C8—C9—C10	0.0 (5)
N4—Cu1—O1—C1	−149.3 (2)	N3—C8—C9—C10	−179.6 (3)
O1^i^—Cu1—O1—C1	−60.5 (2)	C8—C9—C10—C11	−0.3 (5)
O1—Cu1—O2—C4	−50.3 (5)	C9—C10—C11—C12	0.2 (5)
N1—Cu1—O2—C4	5.74 (19)	C10—C11—C12—C7	0.1 (5)
N4—Cu1—O2—C4	−175.98 (19)	C8—C7—C12—C11	−0.4 (5)
O1^i^—Cu1—O2—C4	95.49 (19)	S2—C7—C12—C11	178.9 (2)
Cu1—O1—C1—C16	160.3 (2)	C1—C2—C13—C14	0.9 (5)
Cu1—O1—C1—C2	−22.8 (4)	C3—C2—C13—C14	−177.6 (3)
O1—C1—C2—C13	179.9 (3)	C2—C13—C14—C15	1.4 (5)
C16—C1—C2—C13	−3.2 (4)	C13—C14—C15—C16	−1.3 (6)
O1—C1—C2—C3	−1.7 (5)	C14—C15—C16—C1	−1.1 (5)
C16—C1—C2—C3	175.3 (3)	O1—C1—C16—C15	−179.6 (3)
N2—N1—C3—C2	−178.7 (3)	C2—C1—C16—C15	3.3 (5)
Cu1—N1—C3—C2	7.5 (4)	C21—N4—C17—C18	−1.3 (4)
C13—C2—C3—N1	−171.6 (3)	Cu1—N4—C17—C18	177.0 (2)
C1—C2—C3—N1	10.0 (5)	N4—C17—C18—C19	−0.4 (5)
Cu1—O2—C4—N2	−5.4 (4)	C17—C18—C19—C20	2.0 (5)
Cu1—O2—C4—C5	174.4 (2)	C18—C19—C20—C21	−1.9 (5)
N1—N2—C4—O2	0.7 (4)	C17—N4—C21—C20	1.4 (4)
N1—N2—C4—C5	−179.1 (3)	Cu1—N4—C21—C20	−176.9 (2)
O2—C4—C5—S1	170.0 (2)	C19—C20—C21—N4	0.2 (5)
N2—C4—C5—S1	−10.2 (4)		

Symmetry codes: (i) −*x*+3/2, *y*, *z*−1/2.
